# TLR4 and pSTAT3 Expression on Circulating Tumor Cells (CTCs) and Immune Cells in the Peripheral Blood of Breast Cancer Patients: Prognostic Implications

**DOI:** 10.3390/cancers14041053

**Published:** 2022-02-18

**Authors:** Maria A. Papadaki, Alexia Monastirioti, Christina A. Apostolopoulou, Despoina Aggouraki, Chara Papadaki, Kleita Michaelidou, Maria Vassilakopoulou, Katerina Alexakou, Dimitrios Mavroudis, Sofia Agelaki

**Affiliations:** 1 Laboratory of Translational Oncology, School of Medicine, University of Crete, 71110 Heraklion, Greece; papadaki_maria1@yahoo.gr (M.A.P.); monasal91@gmail.com (A.M.); christinapostol@yahoo.gr (C.A.A.); daggouraki@yahoo.co.uk (D.A.); chapapadak@uoc.gr (C.P.); mkleita@gmail.com (K.M.); marvasila@uoc.gr (M.V.); wj19030@bristol.ac.uk (K.A.); mavroudis@uoc.gr (D.M.); 2 Department of Medical Oncology, University General Hospital of Heraklion, 71110 Heraklion, Greece

**Keywords:** toll-like receptor 4 (TLR4), phosphorylated signal transducer and activator of transcription protein 3 (pSTAT3), circulating tumor cells (CTCs), peripheral-blood mononuclear cells (PBMCs), immune cells, breast cancer, cancer inflammation, immune evasion, peripheral immune response, liquid biopsy

## Abstract

**Simple Summary:**

Toll-like receptor 4 (TLR4) and phosphorylated signal transducer and activator of transcription protein 3 (pSTAT3) play a prominent role in cancer inflammation and anti-tumor immune response, and their therapeutic targeting is considered a promising strategy for the management of breast cancer (BC). We herein hypothesized that these immunomodulatory molecules may be involved in peripheral tumor-immune crosstalk and could provide valuable prognostic information. Our results provide first evidence that the expression of TLR4 and pSTAT3 on circulating tumor cells (CTCs) and immune cells of BC patients might play a role in peripheral anti-tumor response and metastatic progression, and could be associated with patient outcomes.

**Abstract:**

TLR4 and pSTAT3 are key players in cancer inflammation and immune evasion; however, their role in the peripheral blood (PB) is largely unexplored. Herein we evaluated their expression in the circulating tumor cells (CTCs) and peripheral-blood mononuclear cells (PBMCs) of patients with early (*n* = 99) and metastatic (*n* = 100) breast cancer (BC). PB samples obtained prior to adjuvant and first-line therapy, were immunofluorescently stained for Cytokeratins/TLR4/pSTAT3/DAPI and analyzed via Ariol microscopy. TLR4+ CTCs were detected in 50% and 68% of early and metastatic CTC-positive patients, respectively, and pSTAT3+ CTCs in 83% and 68%, respectively. In metastatic patients, CTC detection was associated with a high risk of death (HR: 1.764, *p* = 0.038), while TLR4+ CTCs correlated with a high risk of disease progression (HR: 1.964, *p* = 0.030). Regarding PBMCs, TLR4 expression prevailed in metastatic disease (*p* = 0.029), while pSTAT3 expression was more frequent in early disease (*p* = 0.014). In early BC, TLR4 expression on PBMCs independently predicted for high risk of relapse (HR: 3.549; *p* = 0.009), whereas in metastatic BC, TLR4+/pSTAT3− PBMCs independently predicted for high risk of death (HR: 2.925; *p* = 0.012). These results suggest that TLR4/pSTAT3 signaling on tumor- and immune-cell compartments in the PB could play a role in BC progression, and may hold independent prognostic implications for BC patients.

## 1. Introduction

According to the “cancer immunoediting” theory, newly arising tumors are recognized and destroyed by the immune system. However, malignant cells can exploit different mechanisms to escape from immune surveillance, thus driving the development and growth of a clinically detectable tumor, and subsequently the formation of metastasis [1]. The overcoming of tumor immune evasion formed the basis for the development of different immunotherapy strategies that have already been introduced in clinical practice for the treatment of solid tumors, including triple-negative breast cancer (BC) [2,3]. Cancer-associated inflammation represents another hallmark of cancer, which contributes to genomic instability, epigenetic modification, and the proliferation and dissemination of tumor cells [4]. Studies of the last two decades converge on the existence of a link between chronic inflammation and immune malfunctioning within the tumor microenvironment (TME) [5,6]. Two molecules with a key role in both cancer-associated inflammatory response and immune suppression are toll-like receptor 4 (TLR4) [7] and signal transducer and activator of transcription protein 3 (STAT3) [8].

Toll-like receptors (TLRs) commonly recognize different molecules of microbial origin and trigger upregulation of inflammatory cytokines through cytoplasmic signaling [9]. More specifically for TLR4, its stimulation results in MyD88-dependent activation of NF-κB and MAPK among other pathways, thus, inducing a series of inflammatory cytokines and pro-survival factors [10]. TLR4 expression prevails among key cell subsets of innate immunity, such as monocytes, macrophages, neutrophils and dendritic cells (DCs), while it is identified at a lower level on T cells and B lymphocytes [11]. TLR4 is also expressed on tumor cells and holds a prominent role in inflammation-fueled cancer progression and metastasis. In particular, TLR4 promotes tumor cell proliferation, invasion, survival and migration, the induction of epithelial–mesenchymal transition (EMT), the expansion of cancer stem-cells (CSCs), resistance to paclitaxel [12], and immune suppression in the TME [13].

STAT3 plays a critical role in regulating B cells, CD4+ and CD8+ T cells, and macrophages, as well as T regulatory cells (Tregs) and myeloid-derived suppressor cells (MDSCs) [14,15,16]. STAT3 is activated via phosphorylation by JAK kinases, followed by dimerization of phosphorylated proteins (pSTAT3) which translocate to the nucleus to promote the transcription of target genes. The STAT3 signal pathway is the major intrinsic pathway for tumor-promoting inflammation, and has a key role in the impairment of anti-tumor immunity [8,17]. Specifically for BC, STAT3 promotes its survival, proliferation, progression, metastasis and chemoresistance [18]. A growing body of evidence suggests a link between TLR4 and pSTAT3—more particularly, TLR4 signaling activates and cooperates with STAT3, to induce the formation of EMT-like CSCs [19] and to promote tumor growth and immunosuppression [20].

To date, the role of TLR4 and pSTAT3 has mostly been investigated in the TME, whereas their expression in the periphery is largely unexplored. However, anti-tumor immune surveillance is a dynamic process which often varies among the primary tumor and peripheral tissues [21]. To this end, peripheral-blood mononuclear cells (PBMCs), comprised of all the key circulating immune cell subsets of the host, are frequently analyzed for the real-time identification of immune perturbations in cancer patients [22]. On the other hand, circulating tumor cells (CTCs) can be identified in the peripheral blood (PB) of patients with solid tumors, and are crucial precursors of metastasis [23]. Their analysis provides valuable prognostic information on patient outcomes and contributes to the understanding of the metastatic process [24]. CTCs are endowed with enhanced immune evasion capacities to survive in the blood microenvironment, and their analysis is increasingly utilized for the investigation of mechanisms underlying anti-tumor immune response [25]. In this context, we previously demonstrated that CD47 and PD-L1, two putative immune checkpoints involved in tumor escape, are more frequently expressed on CTCs compared to the corresponding primary or metastatic tumor tissues of BC patients, and that these CTC populations predict for poor patient outcomes [26]. In addition, the comparative analysis of PD-L1 expression among PBMCs and tumor-infiltrating lymphocytes (TILs) from primary and metastatic tumors of these patients, further revealed a significant discordance between the blood and tumor tissue compartments [26]. Consequently, combined CTC and PBMC analysis can provide real-time information on the expression of immunomodulatory molecules in the periphery, and might have clinical implications for BC patients.

We herein hypothesized that TLR4 and pSTAT3 may be involved in tumor-immune crosstalk within the blood circulation, and that their assessment in PB could provide insights into BC progression. To this end, in the present study, we analyzed TLR4 and pSTAT3 expression on CTCs and PBMCs from two large cohorts of early and metastatic BC patients. We show, for the first time, that these two immunomodulatory molecules are frequently expressed on CTCs of BC patients, and that they prevail in the triple-negative subtype. In addition, their expression on PBMCs varies among the two disease stages, with TLR4 prevailing in the metastatic stage, and pSTAT3 in early disease. Importantly, the results demonstrate that phenotyping of CTCs and PBMCs according to these immunomodulatory molecules provides independent prognostic information for BC patients. The current study contributes to the current limited knowledge of the role of TLR4 and pSTAT3 in blood circulation, and suggests that their real-time assessment on CTCs and PBMCs could be used for BC prognosis.

## 2. Materials and Methods

### 2.1. Patients

The current study included patients with early (*n* = 99) and metastatic (*n* = 100) BC, who received treatment at the Department of Medical Oncology, University General Hospital of Heraklion, Greece, between 2011 and 2016. Peripheral blood (PB) samples were collected at the baseline of adjuvant and first-line treatment, respectively. Clinical characteristics and follow-up information were prospectively collected. Consecutive patients with available blood samples who met the following criteria were included: the patient had pathologically diagnosed BC; was over 18 years old; had the ability to provide written, informed consent; and had complete clinical and pathological data. BC patients with secondary malignancies or incomplete clinicopathological data were excluded from the study. One patient with metastatic BC was excluded from the survival analysis only, due to drug-related anaphylaxis and death one day after treatment initiation.

### 2.2. Cell Culture

MCF-7, SKBR-3 and MDA.MB.231 breast cancer cell lines were obtained from the American Type Culture Collection (ATCC) and were cultured as previously described [26]. Following mycoplasma testing using the MycoAlert^TM^ assay, cell cytospins were prepared to serve as controls for the immunofluorescence (IF) staining.

### 2.3. CTC Enrichment

The enrichment of CTCs in blood samples was performed as previously described [26]. Briefly, PBMCs were isolated by Ficoll–Hypaque density-gradient centrifugation, and cytospins of 500,000 cells were prepared and stored at −80 °C until use.

### 2.4. Immunofluorescence (IF)

Triple IF staining for Cytokeratins (CKs)/TLR4/pSTAT3 was performed on cell cytospins. Briefly, cells were fixed with PBS/Formaldehyde 3.7% and permeabilized with PBS/Triton X-100 0.1%. A phospo-STAT3 antibody (Y705) (1:25) (R&D Systems, Minneapolis, MN, USA) was incubated for 1 h, at room temperature (RT), followed by the corresponding secondary antibody, Alexa fluor 555 Anti-Rabbit (1:300), incubated for 45 min at RT. An Alexa Fluor 647-conjugated ΤLR4 antibody (1:50) (Clone: mouse 76B357.1, Novus Biologicals, LLC, Centennial, CO, USA) was incubated overnight at 4 °C. Two different Alexa Fluor 488-conjugated antibodies for CKs (Clones: mouse AE1/AE3 (1:100), Thermo Fisher Scientific, Waltham, MA, USA) and mouse C11 (1:200) (Novus Biologicals) were also included in the overnight incubation, as previously described. Cell nuclei were detected using DAPI antifade (Invitrogen).

### 2.5. Evaluation of TLR4 and pSTAT3 Expression in BC Cell Lines

Cytospins of SKBR-3, MCF-7, and MDA.MB.231 were stained for TLR4 and pSTAT3. All cell lines expressed pSTAT3 in the majority of cells, whereas TLR4 was mainly expressed in MDA.MB.231 cells, which were, therefore, selected to serve as controls for TLR4 and pSTAT3 expression on patient samples (Figure S1). As described in our previous reports, 4 cytospins of MDA.MB.231 cells were included in each separate IF staining performed for patient samples, in order to serve as positive and negative controls for the respective markers [26,27,28]. The intensity of each marker was measured using the Ariol microscopy system (Genetix, New Milton, UK), as previously described [26,27,28].

### 2.6. Evaluation of TLR4 and pSTAT3 Expression in CTCs and PBMCs

A total of 1 × 10^6^ PBMCs (two slides) per patient (total number of slides: *n* = 398) were stained for CK/TLR4/pSTAT3, and analyzed using the Ariol microscopy system, as previously described [26,27,28]. The expression of CKs was used to distinguish CTCs (CK-positive cells) from PBMCs (CK-negative cells). The detection of at least one intact, nucleated cell, positive for CKs, was used to define patient positivity for CTCs, as previously described [26,27,28,29,30].

TLR4 and pSTAT3 expression on CTCs and PBMCs was assessed at the single-cell level using MDA.MB.231 cells as control. The detection of at least one CTC positive for a particular phenotype was used to define patient positivity, as previously described [26,27,28,29,30]. The expression of the two markers was evaluated on 1000 PBMCs in randomly selected microscopy vision fields; two different cut-offs, any (≥0%) or mean expression, were used to define patient positivity as previously described [26]. The analysis was performed by two individual observers (A.M. and C.A.A.), who were blinded to each other’s findings and patients’ clinical data.

### 2.7. Statistical Analysis

Statistical analyses were performed using IBM SPSS Statistics version 20. A Fisher’s exact test and Mann–Whitney U test were used to investigate possible correlations between CTCs and distinct CTC, or PBMC phenotypes and clinicopathological characteristics. The disease-free survival (DFS), progression-free survival (PFS) and overall survival (OS) were calculated as previously described [26], and their association with different parameters was evaluated using Kaplan–Meier and Cox regression analyses. The variables with statistical significance in the univariate Cox regression analysis were included in a multivariate Cox proportional hazards regression model. *p*-values were considered statistically significant at the *p* < 0.05 level. Considering that the study is a small-cohort hypothesis-generating exploratory study, no correction for multiple analyses was performed.

## 3. Results

### 3.1. Patient and Disease Characteristics

The patient and disease characteristics of patients with early and metastatic BC are summarized in Table 1 and Table 2, respectively. Among the 99 early-BC patients, 18 relapses and 13 deaths were recorded at the time of analysis (median DFS and OS were not reached; NR). The median follow-up time for early-BC patients was 92.6 months (95%CI: 88.8–96.5). Among the 99 metastatic BC patients who were eligible for survival analysis, 84 had progressed (median PFS: 12.8 months; 95% CI: 11.1–14.6) and 76 had died (median OS: 32.7 months; 95%CI: 26.8–38.7) at the time of analysis.

### 3.2. TLR4 and pSTAT3 Expression on CTCs

CK+ CTCs were detected in 6/99 (6.1%) and in 19/100 (19%) of patients with early and metastatic BC, respectively (*p* = 0.002). More particularly, CTCs were identified in 12/72 (16.7%) and in 7/28 (25%) of patients with recurrent and de novo metastatic disease, respectively.

**Figure 1:**
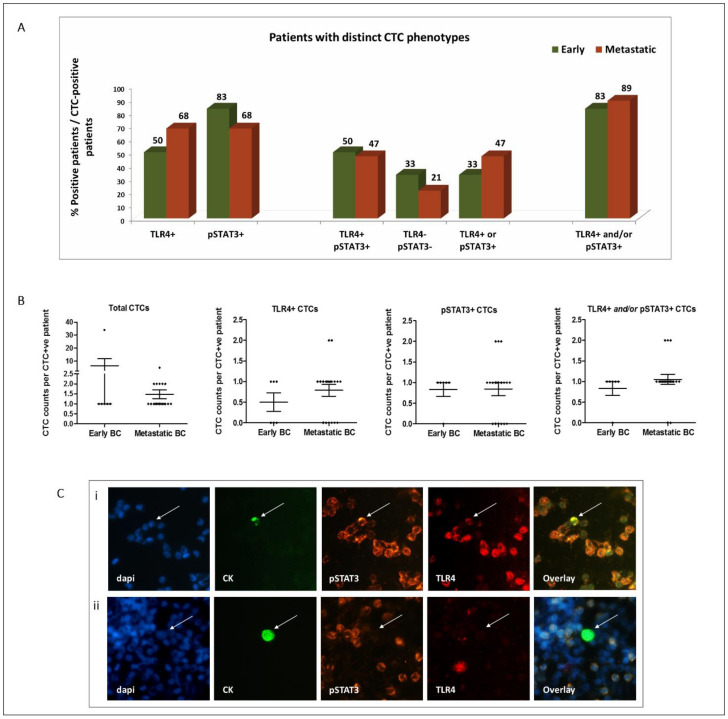
Distribution of TLR4 and pSTAT3 expression on CTCs of patients with early (*n* = 99) and metastatic (*n* = 100) BC: (**A**) percentage of patients harboring distinct CTC phenotypes among CTC-positive patients; (**B**) distribution of CTCs and of distinct CTC subsets among early (*n* = 6), recurrent (*n* = 12) and de novo metastatic (*n* = 7) CTC-positive patients (scatter dot plots, lines correspond to mean values; error bars: standard error of mean (SEM)); (**C**) representative images of TLR4 and pSTAT3 staining on CTCs (CK-positive cells—arrows) and PBMCs (CK-negative cells) identified in the peripheral blood of BC patients. CTCs with distinct phenotypes are shown: (i) pSTAT3+/TLR4+ and (ii) pSTAT3−/TLR4−. DAPI for cell nuclei (blue), CK (green), pSTAT3 (orange) and TLR4 (red). Ariol microscopy system—200×.

TLR4+ CTCs were detected in 50% and 68% of early and metastatic CTC-positive patients, respectively, while pSTAT3+ CTCs were evident in 83% and 68% of patients, respectively (Figure 1A). In addition, CTCs co-expressing the two molecules (TLR4+/pSTAT3+) were frequently detected in both early and metastatic patients (in 50% and 47%, respectively), while positivity for any marker (TLR4+ and/or pSTAT3+) was confirmed in 83% and 89% of patients, respectively (Figure 1A).

The absolute numbers of CTCs and of distinct subsets identified in each cohort are shown in Figure 1B and in Table S1. Notably, all the CTCs identified in the de novo metastatic setting were of the TLR4+ and/or pSTAT3+ phenotype (Table S1). Representative images of distinct CTC phenotypes are shown in Figure 1C.

### 3.3. TLR4 and pSTAT3 Expression on PBMCs

**Figure 2:**
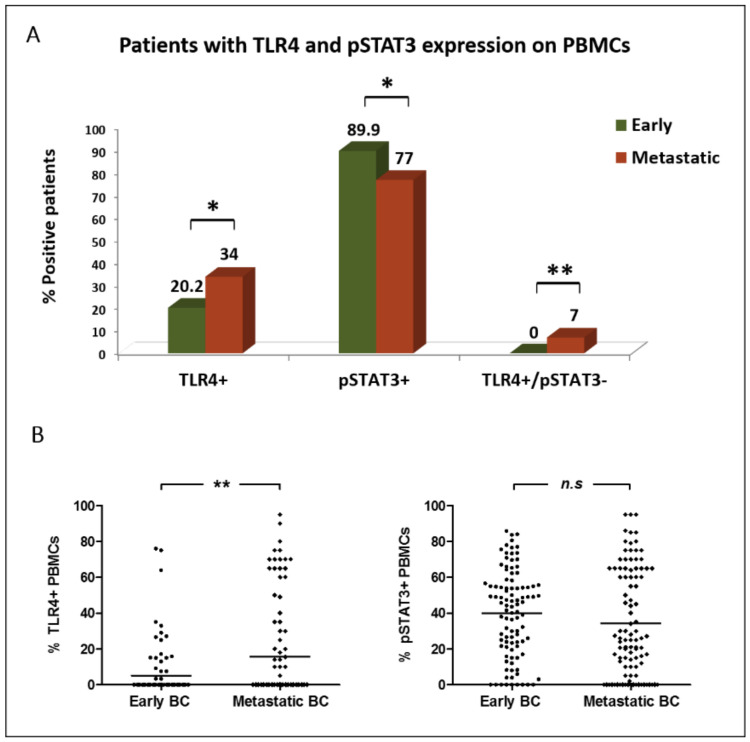
Distribution of TLR4 and pSTAT3 expression on PBMCs of patients with early (*n* = 99) and metastatic (*n* = 100) BC: (**A**) percentage of patients harboring distinct expression profiles on their PBMCs; (**B**) percentage of PBMCs expressing distinct phenotypes (scatter dot plot, lines correspond to mean values). *, ** Statistical significance at the *p* < 0.05 level.

TLR4+ PBMCs were more frequently detected among patients with metastatic BC compared to early BC (34% vs. 20.2% of patients, respectively; *p* = 0.029; positivity was defined as any expression >0%). Metastatic patients also harbored increased percentages of TLR4+ PBMCs (mean % per patient: 15.8% vs. 5.2%; *p* = 0.009) (Figure 2A,B).

On the contrary, pSTAT3 expression on PBMCs was frequently observed in both disease settings; however, it was more prevalent in early than in metastatic BC (89.9% vs. 77% of patients, respectively (*p* = 0.014; cut-off for pSTAT3 positivity: >0%)). A numerically higher percentage of pSTAT3+ PBMCs was also demonstrated in early-stage patients (mean % per patient: 40% vs. 34.3%; *p* = 0.099) (Figure 2A,B).

There was a positive correlation between the percentages of TLR4 and pSTAT3 expression on PBMCs in both early and metastatic disease settings (*p* = 0.001 and *p* = 0.025, respectively; Spearman’s rho correlation). However, notably, PBMCs positive for TLR4 only (TLR4+/pSTAT3−) were detected in metastatic disease only (0% vs. 7% of patients; *p* = 0.007) (Figure 2A).

Representative images of PBMCs expressing TLR4 and/or pSTAT3 are depicted in Figure 1C. Notably, the expression of neither TLR4 nor pSTAT3 on PBMCs was associated with the detection or phenotype of CTCs.

### 3.4. Clinical Relevance of CTCs and of TLR4 and pSTAT3 Expression on CTCs

#### 3.4.1. Early BC

No correlation was observed between the detection of CTCs or distinct CTC subsets and clinicopathological characteristics, or survival outcomes, of early-BC patients.

#### 3.4.2. Metastatic Disease

The detection of TLR4+/pSTAT3+ CTCs prevailed in patients with triple-negative BC over ER+ and/or PR+/HER2− and HER2+ BC settings (in 25% vs. 9.4% vs. 0% of patients, respectively; *p* = 0.031). Notably, all the CTCs identified in the triple negative setting were of the TLR4+/pSTAT3+ phenotype. No other correlations were observed between the detection or phenotype of CTCs and clinicopathological characteristics.

Also, patients experiencing disease progression at the first evaluation of their response to treatment more frequently harbored CTCs at baseline of treatment compared to those with stable disease (SD) or partial response (PR) to treatment (38.9% vs. 23.5% vs. 9.8% of patients, respectively; *p* = 0.035).

**Figure 3:**
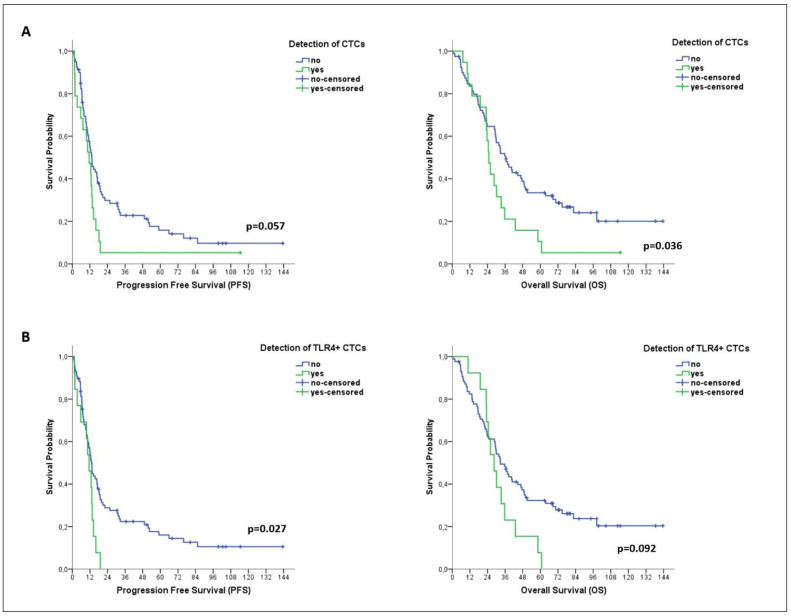
Clinical relevance of CTCs and distinct CTC subsets in patients with metastatic BC. Kaplan–Meier plots for progression-free survival (PFS) and overall survival (OS) of patients with metastatic disease (*n* = 99), based on the detection of total CTCs (**A**) and of TLR4+ CTCs (**B**).

Kaplan–Meier analysis revealed a shorter OS among CTC-positive patients (median OS: 24.9 months vs. 36.5 months; *p* = 0.036) (Figure 3A). Moreover, a shorter PFS was demonstrated for patients harboring the TLR4+ CTC subset (median PFS: 11.4 months vs. 13.1 months; *p* = 0.027) (Figure 3B). No association was shown between pSTAT3 expression on CTCs and patient outcomes.

In univariate analysis, the detection of TLR4+ CTCs was associated with a high risk of progression (HR: 1.964; 95% CI: 1.066–3.617; *p* = 0.030) (Table 3). In multivariate analysis, TLR4+ CTCs emerged as the only factor predicting high risk of progression (HR: 1.859; 95% CI: 1.003–3.447; *p* = 0.049) (Table 3).

### 3.5. Clinical Relevance of TLR4 and pSTAT3 Expression on PBMCs

#### 3.5.1. Early Disease

No correlation was observed between TLR4 or pSTAT3 expression on PBMCs, or patient and disease characteristics (age, menopausal status, histology, or tumor grade, stage and molecular subtype).

**Figure 4:**
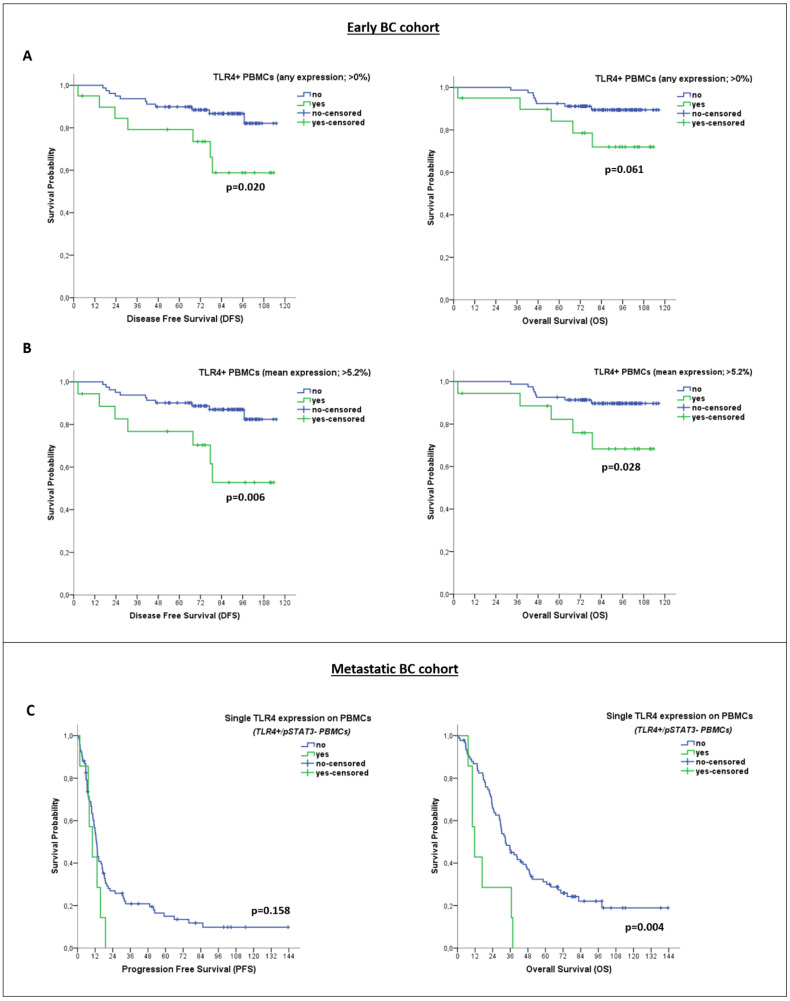
Clinical Relevance of TLR4 and pSTAT3 expression on PBMCs of patients with early and metastatic BC. Kaplan–Meier plots for disease-free survival (DFS) and overall survival (OS) of early-BC patients (*n* = 99), based on TLR4 expression on PBMCs (using different thresholds for positivity, regardless of pSTAT3 expression status) (**A**,**B**). Kaplan–Meier plots for progression-free survival (PFS) and OS of metastatic BC patients (*n* = 99), based on single TLR4 expression on PBMCs (TLR4+/pSTAT3− phenotype) (**C**).

However, Kaplan–Meier analysis revealed significantly reduced survival rates among early-BC patients with TLR4 expression on PBMCs (identified by using any expression (>0%) or mean expression (>5.2%) as thresholds for positivity) (median DFS: NR vs. NR; *p* = 0.020 and *p* = 0.006, respectively, and median OS: NR vs. NR; *p* = 0.061 and *p* = 0.028, respectively) (Figure 4A,B). No association was observed between pSTAT3 expression on PBMCs and patient outcomes.

Univariate cox-regression analysis further confirmed an association between TLR4 expression on PBMCs and high risk for relapse (HR: 3.459; 95%CI: 1.338–8.940; *p* = 0.010) and death (HR: 3.267; 95%CI: 1.068–9.992; *p* = 0.038) (Table 4). In multivariate analysis, TLR4 expression on PBMCs emerged as the only independent factor predicting a high risk of relapse (HR: 3.549; 95%CI: 1.372–9.182; *p* = 0.009) (Table 4).

#### 3.5.2. Metastatic Disease

TLR4 expression on PBMCs was associated with visceral metastases (detected in 42.6% vs. 16.7% of patients with and without visceral metastases, respectively; *p* = 0.013). No other correlations were observed between PBMC phenotypes and patients’ clinicopathological characteristics (age, menopausal status, histology, tumor stage or subtype, or number of disease sites) or response to treatment.

Additionally, isolated TLR4 or pSTAT3 expression on PBMCs was not associated with patient outcomes. However, patients with PBMCs expressing TLR4 only (TLR4+/pSTAT3− PBMCs) showed remarkably reduced OS rates (median OS: 11.7 months vs. 32.7 months; *p* = 0.004) (Figure 4C). As mentioned above, PBMCs of this particular phenotype were exclusively evident in the metastatic disease setting (Figure 2A). Univariate cox regression analysis also revealed an association between TLR4+/pSTAT3− PBMCs and a high risk of death in patients with metastatic disease (HR: 3.061; 95% CI: 1.378–6.796; *p* = 0.006) (Table 3). Multivariate cox regression analysis further confirmed that TLR4+/pSTAT3− PBMCs (HR: 2.925; 95% CI: 1.269–6.743; *p* = 0.012), along with age above median (HR: 1.891; 95% CI: 1.156–3.094; *p* = 0.011) and metastases in more than two systems (HR: 3.061; 95% CI: 1.378–6.796; *p* = 0.006) can independently predict for a high risk of death (Table 3).

## 4. Discussion

TLR4 and pSTAT3 represent major regulators of cancer inflammation and anti-tumor immune response; however, their role in the periphery is largely unexplored. We herein investigated, for the first time, their expression on the tumor and immune-cell compartments in the PB of patients with early and metastatic BC. Results provide first evidence that TLR4 and pSTAT3 are expressed at the CTC level and are associated with the triple-negative BC subtype. Importantly, TLR4 expression on CTCs independently predicts for high risk of disease progression in metastatic BC. Results also show a differential distribution of the two molecules on PBMCs, with TLR4 prevailing in the metastatic stage, in contrast to pSTAT3, which prevails in early disease. Notably, TLR4 expression on PBMCs is the only independent factor predicting high risk of relapse in early BC, whereas the TLR4+/pSTAT3− phenotype of PBMCs independently predicts high risk of death in metastatic patients.

We here demonstrate, for the first time, that TLR4 and pSTAT3 are frequently expressed on the CTCs of BC patients. Previous evidence from preclinical models and tumor tissues support that TLR4 and STAT3 signaling pathways cooperate on cancer cells to promote EMT, stemness, tumor growth, and immunosuppression [19,20]. We have previously demonstrated that the CTCs of BC patients frequently express EMT/stem-like phenotypes, and putative immune checkpoints such as CD47 and PD-L1 [26,31]; consequently our findings provide indications that these mechanisms may also cooperate on BC patients’ CTCs. The present study further demonstrates an association between TLR4 and pSTAT3 expression on CTCs and the triple-negative BC subtype. Notably, all CTCs identified in the triple-negative setting were of the TLR4+/pSTAT3+ phenotype. This observation is in line with the general assumption that triple-negative is the most immunogenic BC subtype [3,32], and confirms a growing body of evidence underlining the pivotal role of TLR4 and pSTAT3 in the progression, metastasis, chemoresistance and immune evasion of triple-negative BC [33,34,35,36,37,38]. This finding also corroborates our previous observation that PD-L1 expression predominates on the CTCs of triple-negative BC patients [26,39]. Indeed, both pSTAT3 and TLR4 are important inducers of PD-L1 expression on cancer cells [40,41,42,43]. Taken together, our observations converge on the notion that the CTCs of triple-negative BC patients are endowed with increased immune-evasion capacities, and further support that CTC analysis contributes to our understanding of the biology of triple-negative BC.

Importantly, we here show that the detection and phenotypic analysis of CTCs may have significant prognostic implications for metastatic BC patients. Specifically, CTC detection was associated with high risk of death, while TLR4 expression on CTCs emerged as an independent factor predicting high risk for disease progression. In line with this finding, the adverse prognostic role of TLR4 expression on tumor tissues has been demonstrated in a large meta-analysis of patients with different malignancies [44], which was individually confirmed for BC [45,46]. On the other hand, we did not identify any association between pSTAT3 expression on CTCs and patient outcomes. Controversial data also exist regarding the prognostic role of pSTAT3 expression on BC tissues [47,48,49], which could be associated with the fact that STAT3 activation is a dynamic event, and its role in BC onset and progression is a matter of context and time [50,51]. Although we acknowledge that limitations exist in the interpretation of these results due to the low numbers of CTC-positive patients, they imply that further phenotyping of CTCs could provide complementary prognostic information to that obtained by mere CTC detection [28,39]. Additional studies in larger patient cohorts are required to understand the role of TLR4 and pSTAT3 signaling on CTCs, and to investigate potential therapeutic opportunities emerging from these findings.

TLR4 and pSTAT3 are also expressed in a plethora of immune cells and play a pivotal role in the regulation of the immune microenvironment in BC [52]. We here demonstrate that they are differentially distributed on circulating immune cells among early and metastatic BC. In particular, we show that TLR4 expression on PBMCs predominates in metastatic disease, whereas pSTAT3 is more frequently expressed in the early disease setting. Notably, TLR4+/pSTAT3− PBMCs were detected in metastatic BC only. Most importantly, we show that TLR4 expression on PBMCs of early-BC patients is the only independent factor predicting high risk of relapse. In addition, TLR4+/pSTAT3− PBMCs independently predict high risk of death among patients with metastatic disease. These observations collectively suggest an adverse prognostic role of TLR4 and a positive role for pSTAT3, when expressed on PBMCs of BC patients. This could be associated with previous evidence showing that during inflammatory response, pSTAT3 can restrain TLR4 signal transduction on immune cells, particularly on macrophages [53,54], and that this modulation of TLR4-mediated inflammatory responses via pSTAT3 is dynamic and time-dependent [55]. In line with our observations, TLR4 polymorphisms on PBMCs were more frequently detected in metastatic than early colorectal cancer (CRC) patients [56]. In contrast, a decreased TLR4 expression was reported on natural killers (NKs) from patients with BC or CRC compared to healthy donors [57], which might indicate a different role of TLR4 expression on this specific immune cell subset. In accordance to our findings for pSTAT3, previous evidence show that it was less frequently expressed on circulating CD4+ T cells of BC patients compared to healthy individuals, and that it predicted favorable outcomes [58]. Accordingly, pSTAT3 expression on peripheral-blood CD4+ T cells and Tregs from melanoma patients was correlated with the clinical benefit from adjuvant treatment with PD-1 inhibitor [59]. Notably, pSTAT3 has been recognized as a key regulator of PD-L1 in the immune microenvironment in BC [60]; in this context, the current results corroborate our previous finding that PD-L1 expression also prevails on the PBMCs of early-BC patients [26]. Overall, we here show, for the first time, that TLR4 and pSTAT3 expression on peripheral immune cells provides valuable prognostic information for BC patients, which importantly, can be obtained via the analysis of bulk PBMCs. This approach allows the analysis of the entire peripheral immune cell compartment with reduced cost and technical requirements. Nevertheless, future studies utilizing transcriptomic, and flow cytometric approaches would help to identify specific peripheral immune alterations associated with TLR4 and/or pSTAT3 expression on PBMCs, in order to delineate their role in the peripheral immune response in BC [61,62].

The limitations of our study include the low CTC numbers obtained, probably due to the methodology applied for CTC enrichment (Ficoll density gradient centrifugation) and the small blood volumes analyzed (1 million PBMCs per patient, corresponding to an average of 1 mL peripheral blood). Despite the low yield of this approach compared to other automated CTC-enrichment techniques [27], it has been successfully used to identify CTC populations with clinical significance for BC patients [26,28,29]. The current study failed to confirm the prognostic relevance of CTCs in the early-disease setting, thus implying that approaches with greater performance, such as the Parsortix system, would rather be utilized for CTC analysis in early BC [27]. Another limitation might be that the multiple comparison error rate across the reported statistical analyses was not controlled, since our study is a small-cohort hypothesis-generating exploratory study, and it would probably increase type II errors. In addition, we did not include the hematopoietic marker CD45 in the immunofluorescence panel, due to the limitation of our method in using up to four markers (CKs, TLR4, pSTAT3, DAPI). Additionally, the methodology used here for PBMC phenotyping included the analysis of relatively low numbers of cells (approximately 1000 cells per sample), in comparison to hundreds of thousands of cells that can be analyzed by flow cytometry. Although our method was highly reproducible between individual samples of the same patient, the limited number of PBMCs analyzed could result in an underestimation of molecules that are rarely expressed on circulating immune cells. Nonetheless, our approach makes feasible the parallel assessment of CTCs and PBMCs within the same sample, and their characterization at the single-cell level, through high-resolution imaging via the Ariol microscopy system. 

To summarize, we here show, for the first time, that TLR4 and pSTAT3 are frequently expressed on the CTCs of BC patients, and that their expression prevails in the triple-negative subtype. In addition, their distribution on PBMCs varies among the early and metastatic setting. Importantly, TLR4 expression on CTCs, and especially on PBMCs, provides independent prognostic information for patients with early and metastatic BC. The results indicate that these immunomodulatory molecules may be involved in tumor-immune crosstalk in the periphery. Functional and transcriptomic analyses would help to specify their role in the PB of BC patients. Moreover, the therapeutic targeting of STAT3 or TLR4, in combination with anti-PD-1/PD-L1 inhibitors, demonstrates encouraging results in early clinical trials [52,63,64,65,66,67], and predictive biomarkers are required to rationally incorporate combination immunotherapy strategies into clinical practice. The dynamic changes of immune responses during disease progression underline the need for analyzing peripheral tissues, in addition to the primary tumor tissue, as a source of biomarker discovery [21]. Real-time phenotyping of CTCs and PBMCs according to TLR4 and pSTAT3, along with other immune checkpoints, could serve as a non-invasive tool for the identification of patients who could benefit from these approaches.

## 5. Conclusions

The current study provides first evidence that TLR4 and pSTAT3 signaling on CTCs and PBMCs might play an important role in the peripheral anti-tumor response and metastatic progression of BC. The parallel analysis of CTCs and PBMCs allows the real-time assessment of putative immunomodulatory molecules on the peripheral tumor and immune-cell compartments, and may provide significant prognostic information for BC patients. The prognostic and therapeutic implications of these findings merit further investigation.

## Figures and Tables

**Table 1:** Patient and disease characteristics of patients with early breast cancer (BC).

Early-BC Patients (*n* = 99)	*n* (%)
Age, years; median (range)	55 (32–81)
Menopausal status (MS)	
Pre-menopausal	42 (42.4)
Post-menopausal	55 (55.5)
Unknown	2 (2)
Histology	
Ductal	83 (83.8)
Lobular	11 (11)
Mixed	2 (2)
Unknown	3 (3)
Grade	
I–II	44 (44.4)
III	43 (43.4)
Unknown	12 (12.1)
Stage	
I	22 (22.2)
ΙΙ	60 (60.6)
IΙΙ	14 (14.1)
Unknown	3 (3)
Subtype	
ER+ and/or PR+/HER2−	71 (71.7)
HER2+	17 (17.2)
Triple-negative	10 (10.1)
Unknown	1 (1)
Adjuvant treatment ^a^	
Chemotherapy	98 (99)
Hormone therapy	76 (76.8)

Abbreviations: ER—estrogen receptor; PR—progesterone receptor. ^a^ Patients with HER2-positive disease received trastuzumab.

**Table 2:** Patient and disease characteristics of patients with metastatic breast cancer (BC).

Metastatic BC Patients (*n* = 100)	*n* (%)
Age, years; median (range)	59 (29–84)
Menopausal status	
Pre-menopausal	29 (29)
Post-menopausal	69 (69)
Unknown	2 (2)
Histology	
Ductal	83 (83)
Lobular	9 (9)
Mixed	5 (5)
Unknown	3 (3)
Stage at diagnosis	
I–III	72 (72)
IV	28 (28)
Subtype	
ER+ and/or PR+/HER2−	64 (64)
HER2+	24 (24)
Triple-negative	12 (12)
Visceral metastases	
Yes	68 (68%)
No	30 (30%)
Unknown	2 (2%)
Disease sites	
1–2	63 (63)
>2	34 (34)
Unknown	3 (3)
First-line treatment ^a^	
Chemotherapy	88 (88)
Hormone therapy	12 (12)
Response to treatment at first evaluation	
Partial response (PR)	41 (41)
Stable disease (SD)	34 (34)
Progressive disease (PD)	18 (18)
Non-evaluable (NE)	7 (7)

^a^ Patients with HER2-positive disease received trastuzumab.

**Table 3:** Univariate and multivariate Cox-regression analysis for PFS and OS among patients with metastatic BC (*n* = 99).

Cox Regression Analysis	Progression-Free Survival (PFS)				Overall Survival (OS)			
	Univariate		Multivariate		Univariate		Multivariate	
Covariates	HR (95% CI)	*p* Value	HR (95% CI)	*p* Value	HR (95% CI)	*p* Value	HR (95% CI)	*p* Value
Age (>59)	1.260 (0.815–1.946)	0.299			1.737 (1.102–2.740)	0.018 *	1.891 (1.156–3.094)	0.011 *
Menopausal Status (post vs. pre)	1.180 (0.718–1.941)	0.513			1.435 (0.849–2.425)	0.177		
Stage at diagnosis (III vs. IV)	1.512 (0.927–2.465)	0.098			1.563 (0.928–2.633)	0.093		
Histology (ductal)	1.460 (0.695–3.065)	0.318			1.691 (0.838–3.412)	0.143		
*Molecular subtype of tumor*								
ER+ and/or PR+/HER2−	reference				reference			
HER2+	1.510 (0.882–2.586)	0.133			1.496 (0.851–2.630)	0.133		
Triple-negative	2.309 (1.101–4.841)	0.027 *	1.592 (0.854–2.965)	0.143	1.597 (0.722–3.535)	0.248		
Visceral metastases	1.131 (0.709–1.802)	0.606			1.143 (0.694–1.884)	0.599		
No. of disease sites (>2)	1.350 (0.859–2.123)	0.193			1.689 (1.052–2.710)	0.030 *	2.044 (1.246–3.353)	0.005 *
*PBMC expression (yes* vs. *no)*								
TLR4+ PBMCs	1.029 (0.653–1.622)	0.902			1.029 (0.637–1.663)	0.906		
pSTAT3+ PBMCs	0.948 (0.577–1.558)	0.833			0.850 (0.499–1.449)	0.55		
TLR4+/pSTAT3− PBMCs	1.747 (0.797–3.827)	0.163			3.061 (1.378–6.796)	0.006 *	2.925 (1.269–6.743)	0.012 *
*CTC populations (yes* vs. *no)*								
Bulk CTCs	1.664 (0.980–2.824)	0.059			1.764 (1.031–3.016)	0.038 *	1.750 (0.993–3.087)	0.053
TLR4+ CTCs	1.964 (1.066–3.617)	0.030 *	1.859 (1.003–3.447)	0.049 *	1.677 (0.913–3.079)	0.095		
pSTAT3+ CTCs	1.248 (0.689–2.259)	0.465			1.600 (0.877–2.920)	0.125		
TLR4+ *and/or* pSTAT3+ CTCs	1.464 (0.856–2.502)	0.164			1.594 (0.925–2.746)	0.093		

* Statistical significance at the *p* < 0.05 level. Only variables showing statistical significance in univariate analysis were subsequently included in multivariate analysis, following the one in ten rule (molecular subtype of tumor and TLR4+ CTCs were tested for PFS; age, number of disease sites, TLR4+/pSTAT3− PBMCs, and bulk CTCs were tested for OS.

**Table 4:** Univariate and multivariate Cox-regression analysis for DFS and OS among patients with early BC (*n* = 99).

Cox Regression Analysis	Disease-Free Survival (DFS)				Overall Survival (OS)			
	Univariate		Multivariate		Univariate		Multivariate	
Covariates	HR (95% CI)	*p* Value	HR (95% CI)	*p* Value	HR (95% CI)	*p* Value	HR (95% CI)	*p* Value
Age (above vs. below median)	1.916 (0.740–4.960)	0.18			3.922 (1.078–14.266)	0.038 *	3.359 (0.904–12.477)	0.07
Menopausal Status (post vs. pre)	1.355 (0.524–3.505)	0.531			2.847 (0.782–10.361)	0.112		
Stage (III vs. I/II)	2.753 (1.032–7.342)	0.043 *	2.613 (0.976–6.995)	0.056	2.430 (0.747–7.900)	0.14		
Grade (III vs. I/II)	2.362 (0.802–6.958)	0.531			2.443 (0.631–9.448)	0.196		
Histology (ductal)	1.734 (0.497–6.050)	0.388			1.696 (0.371–7.742)	0.496		
*Molecular subtype of tumor*								
ER+ and/or PR+/HER2−	reference				reference			
HER2+	0.965 (0.274–3.391)	0.955			0.800 (0.101–6.318)	0.833		
Triple-negative	1.300 (0.292–5.783)	0.731			1.423 (0.385–5.262)	0.597		
*PBMC expression (above* vs. *below mean)*								
TLR4+ PBMCs	3.459 (1.338–8.940)	0.010 *	3.549 (1.372–9.182)	0.009 *	3.267 (1.068–9.992)	0.038 *	2.529 (0.812–7.878)	0.109
pSTAT3+ PBMCs	0.920 (0.363–2.333)	0.861			0.496 (0.153–1.612)	0.244		
*CTC populations (yes* vs. *no)*								
Bulk CTCs	2.030 (0.465–8.856)	0.346			2.923 (0.647–13.205)	0.163		
TLR4+ CTCs	1.860 (0.247–14.006)	0.547			3.085 (0.399–23.825)	0.28		
pSTAT3+ CTCs	1.131 (0.150–8.513)	0.905			1.602 (0.208–12.329)	0.651		
TLR4+ *and/or* pSTAT3+ CTCs	1.131 (0.150–8.513)	0.905			1.602 (0.208–12.329)	0.651		

* Statistical significance at the *p* < 0.05 level. Only variables showing statistical significance in univariate analysis were subsequently included in multivariate analysis (stage and TLR4+ PBMCs were tested for DFS; age and TLR4+ PBMCs were tested for OS).

## Data Availability

The data presented in this study are available on request from the corresponding author. The data are not publicly available due to privacy/ethical restrictions.

## Supplementary Materials

The following are available online at https://www.mdpi.com/article/10.3390/cancers14041053/s1, Figure S1: Co-expression of cytokeratins (CKs), TLR4 and pSTAT3 on MDA.MB.231 control cell line; Table S1: Distribution of total CTCs and distinct subsets among CTC-positive patients with early, recurrent and de novo metastatic BC.
