# Supplementary material for: TLR4 and pSTAT3 Expression on Circulating Tumor Cells (CTCs) and Immune Cells in the Peripheral Blood of Breast Cancer Patients: Prognostic Implications
Source: Cancers (Basel). 2022 Feb 18;14(4):1053. doi: 10.3390/cancers14041053 (PMC8869985; doi:10.3390/cancers14041053)
Supplement: Supplementary file 1 [file cancers-14-01053-s001.zip › cancers-1540696-supplementary.pdf]

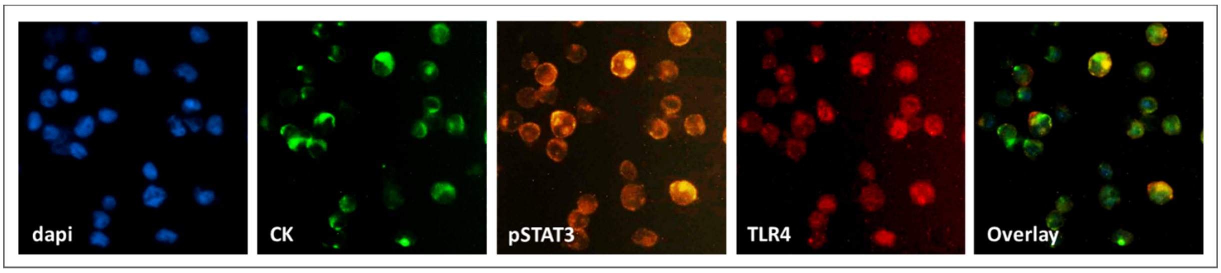

Figure S1: Co-expression of cytokeratins (CKs), TLR4 and pSTAT3 on MDA.MB.231 control cell line

Supplementary Table S1. Distribution of Total CTCs and Distinct Subsets among CTC-positive Patients with Early, Recurrent and De novo Metastatic BC.

CTC-positive Patients	Total CTCs	TLR4+ CTCs	pSTAT3+ CTCs	TLR4+/pSTAT3+ CTCs	TLR4-/pSTAT3- CTCs	TLR4+ or pSTAT3+ CTCs	TLR4+ and/or pSTAT3+ CTCs
Early BC							
# 1	1	0	0	0	1	0	0
# 2	1	1	1	1	0	0	1
# 3	1	0	1	0	0	1	1
# 4	1	1	1	1	0	0	1
# 5	1	1	1	1	0	0	1
# 6	34	0	1	0	33	1	1
Recurrent Metastatic BC							
# 1	1	1	0	0	0	1	1
# 2	1	1	1	1	0	0	1
# 3	2	0	0	0	2	0	0
# 4	1	0	0	0	1	0	0
# 5	1	0	1	0	0	1	1
# 6	1	1	1	1	0	0	1
# 7	1	1	1	1	0	0	1
# 8	1	1	0	0	0	1	1
# 9	2	2	2	2	0	0	2
# 10	5	1	1	1	4	0	1
# 11	1	0	1	0	0	1	1
# 12	2	2	2	2	0	0	2
De novo Metastatic BC							
# 1	1	1	0	0	0	1	1
# 2	1	1	0	0	1	1	2

# 3	1	1	1	1	0	0	1
# 4	1	0	1	0	0	1	1
# 5	1	1	1	1	0	0	1
# 6	2	1	2	1	0	1	2
# 7	1	0	1	0	0	1	1
